# Complementing humanitarian staff care with arts-based initiatives? Viewpoints from in-house mental health professionals

**DOI:** 10.1371/journal.pgph.0006227

**Published:** 2026-07-22

**Authors:** Hannah Strohmeier, Christiane Ganter-Argast

**Affiliations:** 1 Center for Global Health and Institute of International Health, Charité–Universitätsmedizin Berlin, Berlin, Germany; 2 Fakultät Umwelt Gestaltung Therapie, Hochschule für Wirtschaft und Umwelt Nürtingen-Geislingen, Nürtingen, Germany; 3 Universitätsklinikum Tübingen Medizinische Klinik 6, Abteilung für Psychosomatische Medizin und Psychotherapie, Tübingen, Germany; PLOS: Public Library of Science, UNITED STATES OF AMERICA

## Abstract

Over the past decade, research on the mental health of humanitarian staff has gained momentum, prompting organizations to increasingly support and invest in workers’ mental health and well-being. However, many of the existing initiatives—such as one-on-one talk therapy—are grounded in Euro-American traditions. These approaches may not align with the cultural frameworks or preferences of all staff, potentially leaving significant segments of the workforce underserved. Furthermore, challenges related to team cohesion are difficult to address within current staff care structures. Against this backdrop, arts-based initiatives (ABIs) may present a promising complementary approach but remain largely overlooked in humanitarian staff care practice and research. This qualitative study explores the viewpoints of eight in-house mental health professionals (MHPs) currently or previously working for humanitarian and development organisations—a key but hard-to-reach study population—on complementing existing staff care services with ABIs, such as music-making, visual arts, and dance. Data generated through semi-structured online and in-person interviews were analysed using thematic analysis to examine perspectives regarding potential benefits, design considerations, and anticipated challenges related to implementing ABIs in humanitarian workplaces. Three themes reflecting MHPs’ perspectives emerged from the analysis. Overall, MHPs supported introducing ABIs as complementary services. They emphasized what they perceived as the unique potential of these initiatives to enhance well-being, bridge cultural divides, and foster a sense of community, ultimately contributing to a more positive work environment. However, MHPs also underscored the importance of tailoring ABIs to the specific contexts in which they are implemented, requiring carefully designed interventions to prevent unintended negative outcomes. Amongst others, they identified accessibility and buy-in from senior management as key factors for a successful and sustainable rollout. Further research is needed to systematically explore staff preferences, participation factors, integration models, as well as, at a later stage, the effectiveness of ABIs for this occupational group.

## Background

Research on the mental health of humanitarian workers consistently highlights a high prevalence of common mental health problems, including depression, anxiety, burnout, post-traumatic stress disorder, and substance use disorder within this occupational group. Key contributing factors include exposure to traumatic events and chronic stress inherent to the nature of their work [1–6]. More recent studies reveal that intra-organizational issues, such as poor relationships with supervisors and colleagues, as well as racial and gender-based discrimination, also significantly impact staff well-being [7–11]. Recognising these issues, organizations have, over the past decade, intensified their staff care efforts. For example, the United Nations (UN) launched its first Mental Health and Well-being Strategy in 2018 [12], followed by an updated 2024 edition aimed at ensuring accessible support when needed [13]. Similarly, since 2022, the International Committee of the Red Cross has implemented a new staff health strategy that acknowledges the growing pressures on mobile and resident staff, with increased focus on psychosocial support and mental health [14]. Humanitarian non-governmental organisations (NGOs) have also scaled up psychosocial support in recent years [15,16]. In terms of service provision, larger organizations, particularly those within the UN system, generally employ in-house mental health professionals (MHPs) who provide psychoeducation to enhance mental health literacy, as well as counselling and acute care services to staff. Additionally, employees often have access to external therapy covered by employer-provided health insurance. By contrast, smaller organizations, including many NGOs, typically lack in-house services and may instead fund a limited number of individual coaching or psychotherapy sessions per staff member each year provided by contracted external professionals [17].

These developments in staff care are much needed and commendable; the logic behind the services is evidently sound, and the offers certainly resonate with staff. However, significant gaps in support remain across organizations, pointing to areas in need of further improvement. For instance, international staff often have access to a wider array of mental health resources compared to national staff, underscoring disparities in service availability. Consequently, national and international staff frequently express differing preferences and needs concerning staff care [6]. Additionally, stigma associated with using formal mental health services and fears about potential impacts on career prospects still act as barriers, deterring some staff from accessing necessary support [18,19]. Concerns over confidentiality further limit the use of internal psychosocial support services, with many humanitarians expressing a preference for external psychological services, which, however, are often perceived as unsatisfactory, for example due to providers’ limited understanding of the roles of staff and the specific circumstances they operate in [20]. Intra-organizational issues with mental health implications, particularly those related to diversity, equity, and inclusion, are rarely explicitly discussed within organizational mental health strategies, and only a small share of staff members seek support for harm caused by perceived discrimination [9,13]. Lastly, it is important to recognize that many of the psychosocial support services currently offered by organizations—such as one-on-one talk therapy—are grounded in Euro-American traditions of mental health and healing, thereby reflecting another facet of the much-criticised Eurocentrism embedded within humanitarian action [e.g., 21, 22]. These approaches may not align with the cultural frameworks or preferences of all staff, potentially leaving significant segments of the workforce underserved. For example, expressions of trauma vary widely across cultures, requiring the modification of treatments [23,24]. Indeed, research confirms that humanitarian staff care is most effective when adapted to both individual needs and specific cultural contexts [25].

Given the pressing challenges outlined above, it may be time to move beyond the current psychosocial support model and explore complementary approaches that are culturally adaptable, equity-oriented, cost-effective, and capable of alleviating stress and preventing or addressing mental health challenges among humanitarian workers. Ideally, such approaches extend beyond individual wellbeing and foster robust synergies, particularly enhancing team cohesion and nurturing a positive organizational culture, thereby creating a positive feedback loop that further reinforces benefits for staff. At this critical juncture, the arts emerge as a potentially transformative avenue. Arts-based initiatives (ABIs) can be comparably low-treshold and enable three interconnected processes: creating alternative spaces outside dominant structures [26], making power relations visible [27] and analyzable, and centering marginalized voices through non-verbal and symbolic expression [28]. This allows for vulnerability and facilitates the expression of experiences that may otherwise be difficult to articulate, which can be particulary helpful in situations involving trauma or language barriers [29]. When applied in group settings, ABIs are also considered useful for fostering mutual social support among participants. Furthermore, ABIs can be adapted to local forms of expression, religion, and cultural contexts, making them particularly suitable for diverse or underserved populations [30,31]. While they have been successfully implemented by humanitarian organisations to support those affected by crises [32,33], ABIs remain largely overlooked in the formal practice and academic literature surrounding humanitarian staff care.

In 2019, the World Health Organization (WHO) published a much-cited scoping review highlighting the significant role of the arts—performing arts, visual arts, literature, cultural engagement (such as museums and community events), and online or digital arts—in preventing illness, promoting health, and supporting disease management across the lifespan. Arts interventions, the review states, engage multiple modalities, including sensory activation, emotional evocation, cognitive stimulation, and social interaction. These activities, in turn, elicit psychological (e.g., improved coping and emotional regulation), physiological (e.g., reduced stress and enhanced immune function), social (e.g., reduced loneliness and improved social support), and behavioural benefits (e.g., increased physical activity and healthier habits) [34]. The arts have also been associated with fostering prosocial behaviour, group motivation, and collective identity, while promoting empathy and cultural understanding critical for conflict resolution and team cohesion [35].

It should be noted that the WHO report cited above has limitations and has been criticised by some as “uncritical” and “misleading” [36]. In addition, some studies report little or no evidence of improvements in health outcomes associated with ABIs [37]. Nevertheless, despite these critiques and mixed findings, and alongside a growing body of research suggesting potential benefits of the arts for mental health, including in conflict affected contexts [32], ABIs are also increasingly being integrated into occupational health programmes, particularly in high-stress care professions. In this context, activities like creative writing, painting, and choir singing have proven effective in enhancing employee well-being and fostering a positive workplace climate. For instance, a 2023 systematic review of 26 studies found that, while some results were mixed, overall, visual ABIs reduced burnout among healthcare professionals by promoting empathy, connectedness, and tolerance of ambiguity [38]. Similarly, a pilot study in Lithuania showed that participation in workplace art activities improved healthcare workers’ general health and mental well-being, reducing stress, boosting self-esteem, and increasing productivity [39]. Additionally, a UK study on online creativity workshops for COVID-19 crisis staff reported reduced stress and anxiety, alongside increased positive mood and attitudes [40].

The studies above examine ABIs in environments characterised by high workloads and long working hours—conditions comparable to those found in humanitarian settings. However, empirical research on such interventions within humanitarian organizations is scarce, as ABIs are not yet widely implemented across the sector and the experiences of individual initiatives are rarely systematically documented: In an earlier study [6], the first author and colleagues explored desired staff support services based on data collected from national and international staff in South Sudan. Notably, neither the participants nor the research team considered ABIs as part of these services at the time. Similarly, Moclair [18] reports having worked with humanitarian psychosocial staff but provides little detail on the specific context or outcomes. To date, only one peer-reviewed study (based on a review of the English-language literature) appears to address this area in depth: Herbert [41] discusses the use of creative arts therapy techniques, such as creative image-making, movement, and psychodramatic techniques in assessment, supervision, and de-briefing processes to support staff wellbeing policy creation and practice in NGOs in post-conflict Cambodia. They conclude that this model “demonstrated a subsequent reduction in the levels of work-related stress and vicarious trauma” [41] and recommend developing national guidelines for policy and practice.

Despite the research outlined above indicating the potential benefits ABIs might have for humanitarian workers, critical questions remain unanswered. Chief among these are questions regarding the level of interest in, and potential uptake of, such initiatives among humanitarian organizations and their staff. Clarifying these aspects is essential before any meaningful implementation can take place, as this would enable the design of portfolios that are both relevant and effectively utilized. Against this background, two questions guided this research: “What are your thoughts on complementing organizations’ traditional staff care services with ABIs such as dance, music-making, and visual arts?” and “What challenges do you foresee, and how might these be addressed?” These questions were posed to in-house MHPs – a key but hard-to-reach study population – during interviews conducted as part of a broader qualitative research project on humanitarian staff health. MHPs are considered hard to reach because their numbers across the sector are limited, they often carry responsibility for large staff populations, and they work under high workloads often involving significant travel, and strict confidentiality requirements. No ABIs were delivered as part of this study; rather, MHPs were invited to reflect on ABIs from a professional perspective within their organisational roles. Accordingly, this paper does not aim to provide an exhaustive investigation of ABIs, but instead offers an initial exploration of how MHPs perceive their potential role. In this way, it contributes to the necessary preparatory work for assessing whether, and how, ABIs could be integrated into or used to expand existing staff support portfolios.

Before moving to the study, it is important to highlight the distinction between ABIs and art therapy (or arts therapies), and the need for clear conceptual and professional boundaries between both fields [42]: ABIs in health care primarily focus on creativity, relaxation, and social participation and can be facilitated by a variety of professionals, including artists. Art therapy, on the other hand, is a professionally grounded therapeutic discipline and, as a regulated clinical practice, is provided by art therapists—professionals who possess specialized knowledge of art therapy theories, the therapeutic use of art materials, counselling skills, and psychological assessment and crisis intervention [42,43]. This study focuses on ABIs as an accessible, non-clinical entry point to the health-promoting potential of the arts.

## Methods

### Data collection, analysis, and ethics

As mentioned, the data presented in this article were collected as part of a broader research project on humanitarian staff health that the first author led at Charité – Universitätsmedizin Berlin between 2022 and 2024. Eligible participants were MHPs currently or previously working for a humanitarian and/or development organisation in staff care roles, such as stress counsellor, staff counsellor, or psychosocial support coordinator. (Many organisations operate across humanitarian, development, and sometimes peace mandates, using the same in-house MHPs to support all staff.)

To recruit participants, the first author reached out to the UN Headquarters in New York and one organization belonging to the International Red Cross and Red Crescent Movement (hereafter referred to as ‘the Movement’), requesting that they circulate the invitation for study participation among eligible staff. The organization belonging to the Movement supported the study and, in June 2023, sent an email invitation to their MHPs, followed by a reminder email two weeks later. The exact number of MHPs who received this invitation is not known. Three MHPs responded and completed an interview, and two additional MHPs were recruited at a later stage through separate referrals.

The UN did not respond to the request for support. Consequently, the first author applied a snowball sampling approach and used her networks within the UN to recruit participants. Specifically, she reached out to former colleagues and personal contacts who might be able to share the study invitation with eligible MHPs. Due to the nature of snowball sampling, it is not possible to determine how many MHPs were informed about the study through these channels. In addition, she obtained contact details for 13 UN MHPs with whom she had no prior relationship and contacted them directly via WhatsApp. Of these, three did not respond, six initially expressed interest but ultimately did not respond further or were unable to participate (e.g., due to travel, limited internet connectivity, or workload constraints), and four completed an interview. An additional UN MHP was recruited at a later stage through a separate referral.

Semi-structured online and in-person interviews were conducted with all 10 MHPs in the sample over the course of one year (June 2023 to May 2024) to enable an in-depth exploration of professional perspectives, personal observations, and contextual considerations. While the interviews primarily focused on other aspects of staff health, the two questions related to ABIs — “What are your thoughts on complementing organizations’ traditional staff care services with ABIs?” and “What challenges do you foresee, and how might these be addressed?” — were intended to be asked of all participants. However, in keeping with the flexible and participant-led nature of the interviews, time constraints and the flow of individual conversations meant that these questions were ultimately included in eight of the 10 interviews.

All interviews were recorded and transcribed. The first author conducted a thematic analysis of the responses to the two questions on ABIs following the approach outlined by Braun and Clarke [44] and using NVivo software. A deductive approach, using predefined topics derived from the two ABI questions to code and interpret the data was applied; the analysis aimed to examine participants’ perspectives in relation to these thematic areas to capture elements relevant for the practical application of the findings. Codes included, for example, ‘personal views’, ‘previous experience’, ‘benefits’, ‘challenges’, and ‘recommendations.’ During coding, the focus was placed on the explicit, semantic meanings in the data (rather than on latent interpretations) [44], and text was assigned to one code only (‘exclusive coding’) [45].

In this article, the names of participants and organizations are removed, and countries replaced with regions to ensure anonymity. Ethical approval for the study was granted by the Charité Ethics Committee in April 2023 (EA1/081/23). All participants provided informed consent prior to the interview.

### Researcher positionality

The first author is a global mental health researcher at Charité – Universitätsmedizin Berlin with professional and personal connections to the humanitarian sector through consultancy work with the UN. She identifies as a white, cisgender, heterosexual woman. The second author holds a doctorate in education and is a professor of art therapy at HfWU Nürtingen, with experience in clinical art therapy delivery. She also identifies as a white, cisgender, heterosexual woman. We acknowledge that these positionalities may have influenced engagement with participants and aspects of interpretation.

## Results

### Study participants

Most of the eight MHPs who received the questions on ABIs identified as men, were between 30 and 49 years old, and held MA degrees (or equivalent) in the fields of medicine and psychology. Half of the sample worked for the UN, and the other half for an organization within the Movement. Participants held nationalities from countries in the regions of Africa, Asia and the Pacific, Latin America and the Caribbean, and Western Europe and other States (WEO). At the time of the interview, most were serving in duty stations located in WEO (see Table 1).

**Table 1:** Sample characteristics of the mental health professionals interviewed about ABIs.

Gender	Men	6
	Women	2
Age	30-39	4
	40-49	0
	50-59	1
	60+	3
Education	MA	5
	PhD	3
Region of origin	Africa	2
	Asia and the Pacific	1
	Latin America and the Caribbean	2
	Western European and other States	3
Organization	International Red Cross and Red Crescent Movement	4
	United Nations	4
Latest duty station	Africa	1
	Asia and the Pacific	1
	Western European and other States	3
	Other*	3

* Two interviewees had recently retired, and one was in between jobs at the time of the interview. Source: Authors.

### Mental health professionals’ views on arts-based initiatives

The extent of participants’ engagement with the questions on ABIs varied, with some offering brief reflections and others providing more detailed accounts. The data analysis identified three interrelated themes, which are outlined below: Positive potential of ABIs for mental health and organisational climate; strategies for designing and adapting ABIs for the humanitarian workplace; and anticipated challenges and barriers to successful implementation of ABIs (Table 2).

**Table 2:** Overview of themes and examples of verbatims.

Theme	1. Positive potential of arts-based initiatives for mental health and organisational climate	2. Strategies for designing and adapting arts-based initiatives for the humanitarian workplace	3. Anticipated challenges and barriers to successful implementation of arts-based initiatives
**Examples of verbatims**	“That’s brilliant. I think that’s a very important one, that intervention [ABIs] will allow people to express, will give them the opportunity, which is great.”	“I would really, well, be more happy if it’s being offered to all the staff, and not only to staff who can afford, let’s say, 20 USD for a session.”	It “depends on the authorisation from your head of mission (…). You can do [it] in small groups, but if you want to do [it] bigger (…), I think it’s possible, but yeah, the hierarchy needs to be involved (…), needs to authorise that.”
	“Some people brought their painting stuff, and they were painting on a regular basis. I think it’s excellent.”	“I would really believe in, let’s say, finding a (…) [local] artist giving art classes to those who are interested.”	“So you need to convince them to invest their time. So, you would have to put it in a way—like what are the benefits, what are you taking from [it].”
	“This is one of the best ways to bring people to another, different common space.”		“So we cannot provide dancing, for example, in every country, [in] some countries, it’s very difficult. But it could be nice too, because it’s a way you can really get in contact with another person directly.”

Source: Authors.

#### Theme 1: Positive potential of arts-based initiatives for mental health and organisational climate.

Most MHPs reacted positively—some even enthusiastically—to the idea of systematically introducing ABIs in staff care portfolios. They noted that such complementary initiatives would help individuals articulate their experiences and explore their inner worlds more deeply. As one MHP put it: “That’s brilliant. I think that’s a very important one, that intervention [ABIs] will allow people to express, will give them the opportunity, which is great” (Participant 5). The communal and inclusive nature of ABIs was also frequently mentioned as a specific benefit. MHPs considered them attractive to diverse groups of staff across teams, departments, and the organization, fostering team cohesion. Additionally, some MHPs had already had indirect or direct experiences with similar initiatives in their professional lives within humanitarian organizations or roles in the healthcare sector elsewhere. During the interviews, they highlighted the positive potential of ABIs based on these experiences. For example, one MHP noted that in a hardship duty station in Africa, some staff brought painting supplies, which, in their view, was a great idea: “Some people brought their painting stuff, and they were painting on a regular basis. I think it’s excellent” (Participant 3). Another participant described a staff retreat in a different African hardship duty station, noting that incorporating music, games, and painting into team-building activities significantly improved moods, highlighting that “this is one of the best ways to bring people to another, different common space” (Participant 8). Additionally, one interviewee recounted a more formalized experience in a hardship duty station in the AP region where, for security reasons, staff were not permitted to leave the UN compound. Noting that international staff lacked awareness of the local culture and often held misconceptions about the population, they explained: “If the international staff cannot go (…) out to the [city] to see, we can bring [the city] in the UN compound” (Participant 1). This was achieved through arts, writing, painting exhibitions, and music, creating opportunities for cultural exchange. In the scenario this MHP described, the arts served multiple purposes that extended beyond psychosocial benefits. They looked back fondly on this initiative, concluding with excitement that it was “very nice.”

#### Theme 2: Strategies for designing and adapting arts-based initiatives for the humanitarian workplace.

The field of ABIs is vast, offering a variety of implementation options. However, the unique contexts of humanitarian settings necessitate tailored approaches to ensure effectiveness. At the same time, these contexts provide numerous opportunities to harness the wide-ranging impacts ABIs can have. As a result, some interviewees delved deeper into the design and implementation process in their answers. For example, one MHP saw the arts as an activity with a strong social component and suggested using them to foster a sense of community beyond the immediate workplace. They emphasized that, like sports, the arts could connect the wider humanitarian system on-site through “competition, but in a healthy way” (Participant 2). By capitalizing on the strong work ethic and competitive spirit of their UN colleagues, they hypothesized that organizing events beyond the boundaries of a single office could create unity within and between teams. Furthermore, one participant working at their organization’s headquarters reflected on the physical infrastructure of their workplace, which they perceived as “so dry, so cold” and “lacking a space to be together” (Participant 4). They suggested using the unique power of the arts to create a friendlier work environment, specifically one that offers spaces for people to connect.

Considering the uptake of ABIs, one MHP recommended to consider pricing, advocating for initiatives that are accessible to all staff, regardless of their financial situation: “I would really, well, be more happy if it’s being offered to all the staff, and not only to staff who can afford, let’s say, 20 USD for a session” (Participant 3). This interviewee also highlighted the need to avoid labelling the initiatives as ‘art therapy’ and instead refer to them as ‘art services’, for example. They explained that art therapy was challenging to implement because, in their perception, it evokes “an art therapist working in a mental health institution with people of different continents, with severe health problems, mental health problems.” Using different language for services that are “not therapy by a skilled therapist,” however, was seen as promising - for instance, in strengthening the social fabric: “I would really believe in, let’s say, finding a (…) [local] artist giving art classes to those who are interested” (Participant 3).

Lastly, one interviewee fondly recalled a highly successful collaboration with an arts school during a previous role at a psychiatric hospital in an African country. This partnership proved mutually beneficial: the art school supplied materials and supported patients in various art forms, while the hospital granted them permission to collect data and publish their research. Reflecting on this experience, they proposed replicating such a collaborative setup within the humanitarian sector: “I mean, it was a very good deal. If we can do something like that in the UN, I believe it will be very, very important” (Participant 1). Looking ahead to the future of staff care, this interviewee concluded with broad but excited remarks that underscored the importance of accessibility once more: “In every mission, there should be some musical tools, so people could play for example. Or they should be painting or photographing, whatever, we should encourage this, but provide and facilitate it also” (Participant 1).

#### Theme 3: Anticipated challenges and barriers to successful implementation of arts-based initiatives.

Among all participants, only one MHP expressed hesitation about the introduction of ABIs in the current state of the humanitarian sector. Referring to their own organization, they argued that the sector had not yet reached that level of advancement: “No, no, no, we’re not ready. We’re not there yet” (Participant 6). When asked why, they explained that many organizations still do not fully understand the importance of mental health and the urgent need for investments in prevention. They added that the idea of introducing ABIs is conceptually more advanced than what their organization is currently prepared to embrace.

While the other participants were generally less concerned, they did highlight several challenges and barriers – structural, cultural, and personal – that should be considered to ensure the successful implementation of ABIs. One significant challenge brought up in this context was the need to obtain approval from the head of mission/office: their openness to creative solutions was perceived as crucial, as it can either hinder the initiative from the start or pave the way for its success. As one participant put it: it “depends on the authorisation from your head of mission (…). You can do [it] in small groups, but if you want to do [it] bigger (…), I think it’s possible, but yeah, the hierarchy needs to be involved (…), needs to authorise that” (Participant 8). Additionally, interviewees emphasized the necessity for broader institutional buy-in, particularly concerning the sustainability of initiatives and the significance of long-term support. In this context, one interviewee stressed the importance of ensuring that ABIs are not merely a one-off intervention, which risks having minimal impact. Instead, they highlighted the need for an institution that embraces ABIs and mainstreams them in a systematic way. Another participant emphasized the same point by citing the practice of initial pilot projects as an example, noting a concern that these might not result in sustained efforts: “I fear that, well, there will be pilots, and then afterwards, there won’t be anything” (Participant 3). Furthermore, one participant discussed sustainability from the perspective of participation and engagement. They pointed out that, typically, people often respond they are too busy for that. Thus, they stated, a good marketing strategy was crucial to clearly communicate the benefits and encourage participation: “So you need to convince them to invest their time. So, you would have to put it in a way—like what are the benefits, what are you taking from [it]” (Participant 4).

Another challenge identified by interviewees was navigating cultural differences and sensitivities. While ABIs offer the advantage of providing opportunities to learn about other cultures and enhance team cohesion in multi-cultural settings, activities that are acceptable in some countries may not be suitable in others. For example, one interviewee mentioned dancing, highlighting the need to respect local gender norms, which might necessitate separating men and women during such activities. However, they emphasized that when these cultural specifics are carefully considered, dancing can be “a fantastic way to put people together and forget about differences” (Participant 8). Another interviewee also mentioned dancing as an example: “So we cannot provide dancing, for example, in every country, [in] some countries, it’s very difficult. But it could be nice too, because it’s a way you can really get in contact with another person directly” (Participant 1).

Lastly, one interviewee expressed a caution regarding the overall perception of ABIs. They warned that these interventions might not be seen as inclusive enough, particularly by those who are most vulnerable, such as staff facing racial or other forms of discrimination in the workplace. Their concern was that these individuals might feel ABIs are insufficient and merely used as a token gesture, warning that they could think, “oh, look at them. They are treating us like this, and then they ask us to go and participate” (Participant 5).

## Discussion

MHPs are embedded in organisational structures and bring professional backgrounds in mental health-related fields. It remains unclear to what extent their perspectives reflect broader staff experiences and preferences, and their responses should be interpreted in this light. Within this context, MHPs expressed overall strong support for introducing ABIs as complementary services to enhance staff care, particularly in hardship duty stations where conditions are harsh and recreational opportunities scarce. They emphasized, drawing from their experience or foresight, what they perceived as the unique potential of these initiatives to enhance well-being, bridge cultural divides, and foster a sense of community and teamwork, ultimately contributing to a more positive work environment. However, MHPs also underscored the importance of tailoring ABIs to the specific contexts in which they are implemented, requiring carefully designed interventions to prevent potential unintended negative outcomes. These remarks align with Moclair’s [18] conclusion that, based on over two decades of experience as an art therapist and facilitator in humanitarian settings, building a foundational understanding of and confidence in ABIs is essential prior to implementation. The example of dancing raised by MHPs further illustrates that in the specific context of humanitarian organisations, this requires facilitators who are not only experienced in delivering ABIs as mental health and wellbeing initiatives, but who also possess strong cultural competence and a sound understanding of the humanitarian system, including its individual and structural dimensions of inequality and discrimination. Facilitators must be able to adapt interventions accordingly to create respectful, inclusive, and empowering spaces for all staff. This is particularly important given evidence that (racial and gender) discrimination is significantly related to poor mental health [46–48]. In sum, comprehensive ethical considerations - including emotional vulnerability and exposure, as well as potential (perceived or implicit) pressure to participate, and the possible adverse consequences this may have for individuals and groups - must be carefully addressed when designing and implementing ABIs.

Beyond this, affordability was emphasised as an important consideration during the interviews. Indeed, from a power-critical perspective, inequalities in financial means—for example between national and international staff—need to be taken into account, and ABIs must, of course, be accessible to all staff members, not only those in more privileged positions; otherwise, existing power imbalances risk being reproduced. Ensuring accessibility may also lead to more successful implementation and higher participation rates [49].

In addition, MHPs perceived securing buy-in from senior management as crucial for a sustainable and successful rollout. Indeed, the WHO recommends “establish(ing) a mandate for mental health at work by obtaining the buy-in of senior leaders” [50], and studies show that leadership support plays and important role in shaping the attitudes of staff towards mental health [51]. While such buy-in can therefore function as an important enabler, this situation may also be viewed as problematic, as the introduction of (in this case complementary) ABIs appears to depend to a large extent on the priorities and openness of individual managers. This finding is noteworthy given that the availability of, and access to, staff care and wellbeing support should ideally not rely on the preferences of specific leaders but be embedded within organisational structures and policies.

Another enabling factor identified by MHPs was framing ABIs using terminology with fewer therapeutic connotations. These statements must be understood in the context of the fact that seeking any type of therapeutic support is still often experienced as stigmatizing [18,19]. This approach also aligns with common practices in non-clinical art therapy, where practitioners frequently adopt more informal and accessible language to reach a broader audience and intentionally choose non-medical, low-stigma settings for group sessions to help counter stigma, shame, and misconceptions [52,53]. Beyond stigma alone, this practice may also reflect the diversity of needs within staff populations. Some individuals experience crises related to challenging personal or professional circumstances that may call for more therapeutic forms of support, whereas others seek opportunities to build social resources or engage in creative activities for relaxation and balance [54]. The challenge, however, lies in the fact that such varied needs often coexist within the same group and should therefore ideally be addressed simultaneously within a single programme setting. Nevertheless, additional (artistic) psychotherapeutic support is, of course, indicated if participation in ABIs evokes distress or reveals underlying psychosomatic or mental health concerns (e.g., trauma-related symptoms).

One MHP described their workplace as dry and cold, wishing for a friendlier, more connected environment, indirectly highlighting the principles of ‘Healing Architecture.’ Defined as “a scientifically developed concept to nurture the physical and mental well-being of people” [55], healing architecture prioritizes human needs by incorporating features such as large windows for natural light, green spaces, and natural materials like wood. Frequently employed in healthcare and rehabilitation facilities to support recovery processes, it is not directly an ABI as defined in this study, but it may integrate artistic elements, such as murals, artwork, or exhibitions [56]. Given the high levels of stress associated with humanitarian work, incorporating elements of healing architecture could be particularly beneficial in regional and headquarter office locations where feasible. While such designs may not always be possible in field settings—especially in crisis environments where offices are often housed in temporary structures or containers—even small adaptations inspired by healing architecture could contribute to a more supportive and calming environment for staff [e.g., 57].

Since little research on ABIs within the humanitarian sector is documented in peer-reviewed format, the study results can barely be discussed within the context of this body of academic literature. As mentioned, Herbert’s study [41] on creative arts therapy for NGO staff working in post-conflict Cambodia seems currently the only journal article. Yet, the reports from MHPs in this study who had prior experience with ABIs, whether in the humanitarian sector or elsewhere, align with Herbert’s positive evaluation. They are also broadly consistent with research showing the efficacy of ABIs in promoting workplace well-being in other high-stress settings [18,38–40], as well as with Moclair’s [18] summary: “Connecting to our innate creative instinct, via creative expression and exploration, especially in groups, is a powerful, non-pathologizing and very accessible means of individual and collective healing.”

Although ABIs can be effective, it is essential to also recognize their limitations and ensure that they are not used as a convenient substitute for deeper, systemic responsibilities: As indicated by one MHP, they should neither replace efforts to address root causes such as poor working conditions and discrimination nor serve as the sole means of therapeutic intervention. Instead, ABIs are most effective when integrated into broader strategies, providing spaces for exploration and reflection [41]. While systemic issues require comprehensive, long-term solutions, ABIs may offer a useful complement to these efforts by providing a creative and constructive means for staff to engage with complex challenges, particularly when other methods prove insufficient. In this context, collaboration between artists, art therapists, and art educators are highly recommended.

This study has limitations that need to be acknowledged. First, participants’ prior exposure to ABIs varied, and the sample of MHPs interviewed was relatively small, reflecting the challenges associated with recruiting members of this specific occupational group (e.g., varying levels of organisational engagement with recruitment efforts, high workloads and frequent work-related travel among MHPs). In addition, formal data saturation was not systematically assessed during data collection, and the findings may not capture the full range of experiences and perspectives among MHPs working for humanitarian/development organisations. Nevertheless, given the scarcity of existing studies on the research topic, the hard-to-reach nature of the population, and continued debate regarding the role of saturation for sample size determination in qualitative research [58,59], this study’s data offer valuable initial, exploratory contributions to both research and practice. Second, although the sampling strategy employed is widely accepted in qualitative research [60], it remains prone to bias, which warrants cautious interpretation of the findings. Third, the questions on ABIs were included as part of a broader research project and therefore were not explored in extensive depth. For example, ABIs encompass a wide range of practices—including dance, music, and visual arts—and it was not always clear from the interviews which forms participants had in mind when sharing their views. That said, participants were given sufficient time to elaborate on their perspectives and considering that ABIs are not yet formally integrated into staff care systems, it is unlikely that more detailed questioning would have produced substantially different insights at this exploratory stage. Lastly, social desirability or professional advocacy may have influenced participants’ responses – in their role as MHPs they may have been inclined to express support for complementary staff care interventions.

Considering the study’s results and their discussion, this article closes with three recommendations for scholars and practitioners: First, screen the grey literature on art therapy and ABIs to determine whether it includes information on work with humanitarian workers. At the same time, systematically document ABIs that are already being used in contexts like staff retreats, and draw on MHPs’ prior experiences to identify best practices, lessons learned, and inform future programs. Second, launch focused pilot studies to gather data on effectively designing and integrating ABIs into staff care programs. For example, large-scale surveys with humanitarian workers can address key questions, including preferred media and types of initiatives, reasons for participation, and optimal frequency, requiring collaboration between researchers and organisations. On this basis, tailored guidance can be developed to support organisations in integrating ABIs into existing staff care portfolios as complementary approaches to preventing and addressing mental health and wellbeing challenges among humanitarian workers. Such guidance would also explicitly address factors known to influence acceptance, sustained uptake, and efficacy, including affordability; the professional background and training of facilitators; and cultural competence, equity, and inclusion. Third, at a later stage, conduct thorough and methodologically rigorous evaluation studies to assess the effectiveness and limitations of ABIs implemented in organisational contexts, and use these findings to ensure that such initiatives are beneficial, ethically sound, and do not cause unintended harm.
